# An Open-Air School

**Published:** 1923-04

**Authors:** 


### AN OPEN-AIR SCHOOL.

AN interesting account of the work done for "the C 3 child" at the open air school, Thackley, in the municipality of Bradford, is given in the "Yorkshire Post":—

At first children sent here for treatment were mainly from the poorest homes. This is no longer so. The boys and girls, selected as the result of medical inspection from all the schools in Bradford—including secondary schools—are representative of every artisan class. No child is admitted here with any sort of infectious disease. Most suffer from anæmia and the ills that follow in its train. Even those children who show definite symptoms of incipient tuberculosis are sent from the ordinary elementary school to the sanatorium. They are not allowed here.

### The Day's Work.

The children are brought in tramcars every day from Bradford :—

On arrival they receive a breakfast of porridge and warm milk. Many are the amusing stories Mr. Norman (the headmaster) can tell of children, whose parents protested that oatmeal was an indignity their home had never known, who within a fortnight were calling for a second large helping of the odious dish. Immediately after breakfast 60 to 70 per cent. of the little people parade for their daily quota of malt and cod liver oil. From then till 12.15 ordinary school lessons take the field—with this difference, that in summer classes are taken in the leafy shade of the trees, and in winter, with remarkably few days excepted, in the shelter of open verandahs. At noon a substantial dinner is served (stews, soups, fish, and suet and milk puddings). After dinner the children cleanse their mouths and brush their teeth, under the supervision and instruction of a teacher. Then handkerchief drill is taken so as to facilitate nasal breathing in the subsequent "rest" time.

### A Stay of Six Months.

This rest is taken in the open air, or, in bad weather, in sheds open on three sides. After the rest, the children have hand-work, dancing, physical exercises, &c., and a cup of hot milk ends the day, and the children start for home :—

Once a week each child receives a shower bath—in a bathroom that is a shining model of cleanliness—and instruction is given daily in the method of clean washing. A well-fitted remedial room is attached to the school in which a specialist in remedial exercises and massage is occupied full time in treating curvature of the spine and allied defects. Every week sees the arrival of new entrants—weary-limbed, pale of face, languid. Every week sees new departures. In the average stay of six months few little bodies are there that have not benefited. Rosy cheeks where once sat the ashen grey of sickness are no uncommon thing. In the last year over three hundred little patients passed through Mr. Norman's hands, well advanced on the road to health.

### HOSPITAL PENSIONS.

WHILE we still await a general scheme of hospital pensions, it is useful to record any examples of the recognition of the principle, since this is bound eventually to express itself in a proper scheme. Thus the Royal Alexandra Hospital for Sick Children, Brighton, has given to two sisters who have left after serving the institution for twenty and twenty-one years, honoraria of £150 each as a mark of appreciation. A pension fund has been started, in fact, and was lately increased by a sum of £2,000. It is thought that the total now available will be enough to provide pensions for the matrons and sisters in future ; but the amount of the pensions is not apparent.

